# Treating periodontitis-a systematic review and meta-analysis comparing ultrasonic and manual subgingival scaling at different probing pocket depths

**DOI:** 10.1186/s12903-020-01117-3

**Published:** 2020-06-25

**Authors:** Xin Zhang, Zixuan Hu, Xuesong Zhu, Wenjie Li, Jun Chen

**Affiliations:** 1 Department of Orthodontics, Xiangya School of Stomatology, Central South University, Changsha, 410008 People’s Republic of China; 2 Hunan Key Laboratory of Oral Health Research, Hunan 3D Printing Engineering Research Center of Oral Care, Hunan Clinical Research Center of Oral Major Diseases and Oral Health, Central South University, Changsha, 410008 People’s Republic of China; 3 National Key Laboratory of Science and Technology for National Defence on High-strength Structural Materials, Central South University, Changsha, 410008 People’s Republic of China; 4 State Key Laboratory of Powder Metallurgy, Central South University, Changsha, 410008 People’s Republic of China; 5 Department of Periodontics, Xiangya Stomatological Hospital, Xiangya School of Stomatology, Central South University, Changsha, 410008 People’s Republic of China

**Keywords:** Subgingival scaling, Ultrasonic therapy, Periodontal pocket, Periodontal debridement, Meta-analysis

## Abstract

**Background:**

Mechanical plaque removal has been commonly accepted to be the basis for periodontal treatment. This study aims to compare the effectiveness of ultrasonic and manual subgingival scaling at different initial probing pocket depths (PPD) in periodontal treatment.

**Methods:**

English-language databases (PubMed, Cochrane Central Register of Controlled Trials, EMBASE, Medline, and ClinicalTrials.gov, by January, 2019) were searched. Weighted mean differences in primary outcomes, PPD and clinical attachment loss (CAL) reduction, were estimated by random effects model. Secondary outcomes, bleeding on probing (BOP), gingival recession (GR), and post-scaling residual dental calculus, were analyzed by comparing the results of each study. The quality of RCTs was appraised with the Cochrane Collaboration risk of bias tool. The GRADE approach was used to assess quality of evidence.

**Results:**

Ten randomized controlled trials were included out of 1434 identified. Initial PPD and follow-up periods formed subgroups. For 3-months follow-up: (1) too few shallow initial pocket studies available to draw a conclusion; (2) the heterogeneity of medium depth studies was so high that could not be merged to draw a conclusion; (3) deep pocket studies showed no statistical differences in PPD and CAL reduction between ultrasonic and manual groups. For 6-months follow-up: (1) too few shallow initial PPD studies to draw a conclusion; (2) at medium pocket depth, PPD reduction showed manual subgingival scaling better than ultrasound. No statistical differences were observed in CAL reduction between the two approaches; (3) for deep initial PPD studies, both PPD and CAL reduction showed manual subgingival scaling better. GR results indicated no statistical differences at medium and deep initial pocket studies between the two methods. BOP results showed more reduction at deep pocket depths with manual subgingival scaling. No conclusion could be drawn about residual dental calculus.

**Conclusion:**

When initial PPD was 4-6 mm, PPD reduction proved manual subgingival scaling was superior, but CAL results showed no statistical differences between the two means. When initial PPD was ≥6 mm, PPD and CAL reductions suggested that manual subgingival scaling was superior.

## Background

Periodontitis is characterized by gingivitis and periodontal tissue destruction resulting in alveolar bone and tooth loss [1]. Periodontitis is the sixth most common disease with a standardized prevalence of 11.2%. It is the primary cause of tooth loss and negatively affects oral health, nutrition, self-confidence, and overall health. It associates with various systemic chronic diseases such as angiocardiopathy and diabetes [2, 3]. The global burden of periodontal disease remains high, which increased by 57.3% from 1990 to 2010 [4].

Dental plaque biofilm is the initial factor of periodontitis, which aggregates to trigger immune responses. This tends to destroy surrounded soft tissues and alveolar bone [3]. The goal of periodontal treatment is to control infection, and remove dental plaque, dental calculus, and endotoxins [5]. It is confirmed that subgingival scaling is an effective non-surgical periodontal therapy. In the early stage, subgingival scaling was practiced with manual scaling. In recent years, ultrasonic subgingival scaling has been applied in periodontal clinic [1, 6–9]. Each technique has advantages and disadvantages. Currently there is no universal protocol or clinical guideline for selecting one technique over another.

Many in-vivo studies did not group by initial probing pocket depth (PPD) when comparing ultrasonic and manual subgingival scaling, but different PPD may greatly influence instrument selection. For example, Beuchat found that when initial PPD < 6 mm, for gingival recession (GR), there was no statistical difference between ultrasonic and manual subgingival scaling (*P* < 0.05). When initial PPD > 7 mm, attachment level (AL) improved more while GR reduced more in using ultrasonic instruments than using manual instruments [10]. It is controversial in terms of when to use manual subgingival instruments and when to use ultrasonic ones.

In this study, we aimed to compare the work effectiveness between ultrasonic and manual subgingival scaling in different initial PPDs. It can provide new evidence for clinical instrument selection and future study.

## Methods

A protocol has been registered at the International Prospective Register of Systematic Reviews (Number CRD42019125067). The content of this article is consistent with the protocol.

### Research question

The focused question was developed in accordance with recognized Patient, Intervention, Comparison, and Outcome (PICO) format: “What’s the difference of the work effectiveness between ultrasonic and manual subgingival scaling in periodontal treatment at different initial PPDs?”

### Selection criteria

#### Study type

In-vivo randomized controlled trials (RCT) that compared manual- and ultrasonic- subgingival scaling in periodontal treatment were included. Studies had to be in English. We classify the initial PPDs into: (1) shallow pocket: PPD ≤4 mm; (2) medium pocket: 4 mm < PPD < 6 mm; or, (3) deep pocket: PPD ≥6 mm. A group of studies in each article could not be simultaneously included in different PPD categories. Teeth with single-roots or multi-roots were all included in the study.

#### Participants

Adults (age ≥ 18) diagnosed with periodontitis unaccompanied by any other oral or systemic disease and not taking antibiotics.

#### Intervention

Meta-analysis sought to eliminate bias caused by different initial PPDs to compare ultrasonic subgingival scaling with manual ones. No distinctions were made between ultrasonic subgingival instrument makers or models. A Gracey scraper was chosen as the manual instrument.

#### Outcomes

A study may have primary and secondary indicators. Each indicator was processed differently. Not all outcomes were consistent with the criteria. The outcomes included appear in tables (Table 1 and 2).

1. Primary outcomes:

**Table 1:** Included studies characteristics (Primary outcome measures)

Study	First Author, Year Outcomes Mean age (±SEM) Female/Male Country	PPD	Interventions	Follow Up	Inclusion criteria	Exclusion criteria	Smoker/Non-smokers ratio
Pilot study on the clinical and microbiological effect of subgingival glycine powder air polishing using a cannula-like jet [15]	- Kargas, K.2015	moderate pockets	ultrasonic instrumentation (Piezonâ, Instrument A, EMS, Nyon, Switzerland), hand instruments (Gracey curettes 3/4, 11/12, 13/14, Hu-Friedy, Chicago, IL, USA)	6 Months	(a) Must have been previously diagnosed with generalized chronic periodontitis (according to American Academy of Periodontology) and successfully treated; (b) Subsequently, entered the supportive treatment phase (SPT), with at least two non-bleeding residual pockets > 4 mm in each quadrant; (c) Have at least 20 natural teeth; (d) Non-smoker; (e) Could not taken an antibiotic, anti-inflammatory medication, corticosteroids or other immunosuppressive drugs during the previous 6 months; (f) Pregnant or lactating women were also excluded from this study.	None	No smoker
	- PPD, CAL						
	- 52.50 ± 9.54						
	- 12/15						
	- Greece						
Er:YAG lasers versus ultrasonic and hand instruments in periodontal therapy: clinical parameters, intracrevicular micro-organism and leukocyte counts [13]	- Malali, E.2012	4-6 mm, > 7 mm	a magnetostrictive ultrasonic scaler (Cavitron Bobcat Pro, Dentsply International Inc., USA), manual periodontal curettes (Gracey, SG # 5/6, 7/8, 11/12, 13/14, Mini Five Gracey SAS # 5/6, 11/12, Hu-Friedy Ins. Co., USA)	3 Months	Patients with generalized periodontal breakdown and who had at least four single-rooted teeth, two moderately deep (probing depth [PD] of 4–6 mm) and two deep pockets (PD ≥ 7 mm) that had no endodontic lesion and no crown, with mobility 0–2, and with bleeding on probing (BOP) were selected.	(a) Periodontal treatment within the last 6 months; (b) Any systemic disease that would influence the periodontal tissues; (c) Antibiotic used within last 6 months; (d) Pregnancy and smoking.	No smoker
	- PPD、CAL						
	- 48.83 ± 7.23						
	- 11/19						
	- Turkey						
Hand instrumentation versus ultrasonic debridement in the treatment of chronic periodontitis: a randomized clinical and microbiological trial [12]	- Ioannou, I.2009	< 4 mm, 4-6 mm, > 6 mm	UD: (EMS Piezon®, EMS, Nyon, Switzerland) with A and P instruments (Swiss InstrumentsPM, EMS) under water irrigation, SRP:Hu-Friedy Gracey Standard Curettes SG 3/4, 11/12, 13/14, After Five® Curettes SAS 3/4, 11/12, 13/14, Hu-Friedy.	3 Months, 6 Months	(a) Existence of a minimum of four sites with PPD 5 mm in at least two quadrants of each of the patients, demonstrating bleeding on probing; (b) No periodontal treatment during the previous 6 months.	(a) Compromised medical condition; (b) Systemic antibiotics during treatment or for the last 3 months; (c) Ongoing drug therapy that might affect periodontal therapy; (d) Requirement for prophylactic antibiotic cover of the patient; (e) Use of chlorhexidine mouthwash or any other antimicrobial agent; (f) Pregnancy for female patients.	SRP: 50% of patients is smoker;
	- PPD, CAL						
	- SRP:49.62 ± 2.07, UD:50.47 ± 2.58						UD: 52.9% of patients is smoker.
	- SRP:50/50 UD:70.6/29.4						
	- Greece						
Non-surgical periodontal treatment with a new ultrasonic device (Vector™-ultrasonic system) or hand instruments a prospective, controlled clinical study [18]	- Sculean, A. 2004	4-6 mm, > 6 mm	VUS: Vector probe, (Durr Dental, Bietigheim-Bissingen,Germany) using straight and curved metal curettes and a polishing fluid (HA particles < 10um) according to the instructions given by the manufacturer, SRP:Hand instruments (Gracey Curettes, Hu-Friedy Co., Chicago, IL, USA).	6 Months	(a) No treatment of periodontitis for the last 2 years; (b) No use of antibiotics for the 12 months prior to treatment; (c) No systemic diseases, and (d) Good level of oral hygiene. As criterion for a good level of oral hygiene a mean plaque index (PI) score < 1 was chosen.	None	Unclear
	- PPD、CAL						
	- 54						
	- 24/14(VUS:10/9; SRP:11/8)						
	-Germany						
Periodontal healing after non-surgical therapy with a modified sonic scaler: A controlled clinical trial [17]	- Christgau, M. 2006	< 4 mm, 4-6 mm, > 6 mm	UD:the modified sonic scaler system SonicFlex 2003 L (KaVo), SRP:Gracey-curettes #1/2, #7/8, #11/12, #13/14, HuFriedy, Chicago, IL, USA.	6 Months, 1 Month (excluded)	All had generalized moderate to progressive chronic periodontitis, but were systemically healthy and had not received systemic antibiotics for at least 3 months before. Each patient had to show at least four teeth per quadrant with a PPD of at least 4 mm.	None	14/6
	- PPD, CAL						
	- 45.6 ± 8.0						
	- 14/6						
	- Germany						
Full-mouth ultrasonic debridement versus quadrant scaling and root planing as an initial approach in the treatment of chronic periodontitis* [14]	- Wennström, J. L. 2005	5-6 mm, 7 mm	UD:EMS Piezon Master 400 with A + PerioSlim tips, water coolant and power setting to 75%; EMS, Nyon, Switzerland, SRP:LM-dental, Turku, Finland.	3 Months, 6 Months	(a) A minimum of 18 teeth; (b) At least eight teeth must show probing pocket depths (PPD) of 5 mm and bleeding on probing (BOP). At least two of these teeth must have a PPD of 7 mm and at additional two teeth, the pockets must measure 6 mm; (c) Unremarkable general health according to medical history and clinical judgement; (d) Female patients must not be pregnant.	(a) Subgingival instrumentation within 12 months prior to the baseline examination; (b) The use of antibiotics within 3 months prior to the start of the study; (c) Compromised medical conditions requiring prophylactic antibiotic coverage; (d) Ongoing drug therapy that might affect the clinical signs and symptoms of periodontitis.	Italy: UD 4/11, SRP 4/10, Sweden: UD 7/10, SRP 6/11
	- PPD、CAL						
	- 25-75 years old, mean age 49.8						
	- 19/22(SRP11/10;UD8/12)						
	- Italy, Sweden						
Effectiveness of ultrasonic instruments in the therapy of severe periodontitis: a comparative clinical-microbiological assessment with curettes [16]	- D’Ercole, S. 2006	≥6 mm	UD:a power-driven mechanism (Vector® System), SRP: the type of manual instuments is unclear	3 Months, 6 Months, 1 Month (excluded)	(a) Positive for diagnosis of mild-to-severe chronic periodontitis; (b) Good general health according to their medical history; (c) Negative for the use of any antibiotic or antiinflammatory drugs within the 3 months preceding the beginning of the study; (d) Negative for periodontal therapy within 1 year preceding the beginning of the study; (e) Experimental sites (test and control) localized in the interproximal position of two different teeth in the same subject (split-mouth design); (f) Probing depth (PD) values equal to or more than 6 mm in the experimental sites; (g) Difference of PD in the experimental sites (test and control) not exceeding 2 mm; (h) Presence of at least ten teeth for each dental arch. Pregnant or nursing females were excluded from the study.	None	No-smoker
	- PPD、CAL						
	- 40.8 ± 3.9						
	- 11/7						
	- Unclear						

*SRP*, scaling and root planing with hand instrument, *UD* ultrasonic debridement, *PPD* probing pocket depth, *CAL* clinical attachment level

*: The data is from two study centers: Italy and Sweden

**Table 2:** Included studies characteristics (Secondary outcome measures)

Study	First Author, Year Outcomes Mean age (±SEM) Female/Male Country	PPD	Interventions	Follow Up	Inclusion criteria	Exclusion criteria	Smoker/Non-smokers ratio
Pilot study on the clinical and microbiological effect of subgingival glycine powder air polishing using a cannula-like jet [15]	- Kargas, K. 2015	Moderate pockets	Ultrasonic instrumentation (Piezonâ, Instrument A, EMS, Nyon, Switzerland), Hand instruments (Gracey curettes 3/4, 11/12, 13/14, Hu-Friedy, Chicago, IL, USA)	6 Months	(a) Must have been previously diagnosed with generalized chronic periodontitis (according to American Academy of Periodontology) and successfully treated; (b) Subsequently, entered the supportive treatment phase (SPT), with at least two non-bleeding residual pockets > 4 mm in each quadrant; (c) Have at least 20 natural teeth; (d) Non-smoker; (e) Could not taken an antibiotic, anti-inflammatory medication, corticosteroids or other immunosuppressive drugs during the previous 6 months; (f) Pregnant or lactating women were also excluded from this study.	None	No smoker
	- GR						
	- 52.50 ± 9.54						
	- 12/15						
	- Greece						
Clinical evaluation of the speed and effectiveness of subgingival calculus removal on single-rooted teeth with diamond-coated ultrasonic tips [20]	- Yukna, R. A. 1997	5-6 mm, 7-8 mm	Hand curets, Plain ultrasonic	Extract the teeth after the treatment	Subjects had moderately deep probing depths (>5 mm in depth), had not received scaling and root planing for at least 6 months prior to the study, and exhibited clinically and/or radiographically evident subgingival calculus on the study teeth.	None	Unclear
	- The mean percent of calculus remaining						
	- Unclear						
	- Unclear						
	- America						
The effectiveness of the Titan-S sonic scaler versus curettes in the removal of subgingival calculus. A human surgical evaluation [21]	- Gellin, R. G. 1986	< 3 mm, 4-5 mm, 6-12 mm	Ultrasonic instrument (Titan-S), Hand instrument (Gracey curette and the McCall’s)	Extract the teeth after the treatment	Exhibit radiographie evidence of subgingival calculus or a clinically detectable ledge of subgingival calculus on at least one interproximal surface per quadrant, and have no systemic disease contraindicating periodontal therapy or the use of local anesthetics.	None	Unclear
	- The percentage of surfaces with residual calculus						
	- Unclear						
	- Unclear						
	- America						
Non-surgical periodontal treatment with a new ultrasonic device (Vector™-ultrasonic system) or hand instruments a prospective, controlled clinical study [18]	- Sculean, A. 2004	4-6 mm, > 6 mm	UD: Vector probe, (Durr Dental, Bietigheim-Bissingen,Germany) using straight and curved metal curettes and a polishing fluid (HA particles < 10um) according to the instructions given by the manufacturer, SRP:Hand instruments (Gracey Curettes, Hu-Friedy Co., Chicago, IL, USA).	6 Months	(a) No treatment of periodontitis for the last 2 years; (b) No use of antibiotics for the 12 months prior to treatment; (c) No systemic diseases; (d) Good level of oral hygiene. As criterion for a good level of oral hygiene a mean plaque index (PI) score < 1 was chosen.	None	Unclear
	- GR, BOP						
	- 54						
	- 24/14(VUS:10/9; SRP:11/8)						
	-Germany						
Periodontal healing after non-surgical therapy with a modified sonic scaler: A controlled clinical trial [17]	- Christgau, M. 2006	< 4 mm, 4-6 mm, > 6 mm	UD:the modified sonic scaler system SonicFlex 2003 L (KaVo), SRP:Gracey-curettes #1/2, #7/8, #11/12, #13/14, HuFriedy, Chicago, IL, USA.	6 Months, 1 Month (excluded)	All had generalized moderate to progressive chronic periodontitis, but were systemically healthy and had not received systemic antibiotics for at least 3 months before. Each patient had to show at least four teeth per quadrant with a PPD of at least 4 mm.	None	14/6
	- BOP, GR						
	- 45.6 ± 8.0						
	- 14/6						
	- Germany						
Influence of fluorescence-controlled Er:YAG laser radiation, the Vector™ system and hand instruments on periodontally diseased root surfaces in vivo [19]	- Schwarz, F. 2006	> 6 mm	UD:ultrasonic system (Vector™,Dürr,Bietigheim-Bissingen,Germany) and a polishing fluid (hydroxylapatite particles <10 μm) was used according to the instructions given by the manufacturer(70% power setting). SRP: Gracey curets (Hu-Friedy Co., Chicago,IL,USA)	Extract the teeth after the treatment	(a) Probing pocket depths (> 6 mm) on at least two aspects (mesio-buccal/mesio-lingual and disto-buccal/disto-lingual) as measured from the gingival margin to the bottom of the pocket; (b) No signs of carious or artificial damage on the root surface; (c) No periodontal root surface treatment within the last 12 months; (d) No root fractures or anatomical abnormalities.	Patients suffering from systemic diseases were excluded from the study.	Unclear
	- The roughness of cementum surface						
	- 44.8						
	- 7/5						
	- Germany						

*SRP*, scaling and root planing, *UD* ultrasonic debridement; GR gingival recession, *BOP* bleeding on probing

PPD and clinical attachment loss (CAL) were the primary outcomes to compare different subgroup outcomes.

(2) Secondary outcomes:

Bleeding on probing (BOP), gingival recession (GR), and post-scaling residual dental calculus were used as measures. These indicators are of interest, but data about them could not be extracted for meta-analysis due to the limited number of studies, their different measurements, and definitions. They were analyzed by comparing the results of each study.

Studies meeting the following conditions were excluded: (1) follow-up in less than 3 months; (2) treatment during follow-up. Cluster trials were not included.

### Search strategy

PubMed, Cochrane Central Register of Controlled Trials, EMBASE, Medline were searched until January, 2019 for relevant studies. The search was performed using a combination of controlled vocabulary and key words (Appendix 1). Only English articles were searched. No time restrictions were imposed.

For potentially eligible studies, we searched ClinicalTrials.gov for prospective trial registers for controlled trials, with publication time up to January, 2019. In addition, the reference lists from the selected articles were checked for further studies qualifying for the review. Only articles written in English were selected. In this process, an eligible study contrasted ultrasonic and manual subgingival scaling, whether or not other methods were compared at the same time. And the initial PPD of each subject in an eligible study could be classified into shallow, medium or deep pocket at the same time.

### Data collection and analyses

#### Study selection and quality assessment

Three review authors (XZ, ZH, and XSZ) independently searched and included eligible studies, using the same search strategy which had been completed and improved. The quality of each study was reviewed and evaluated for 3 times by three authors and relevant data were extracted. When there was a disagreement whether to include or not, a discussion with other authors (JC and WL) was held and an agreement reached on inclusion or exclusion.

A study’s methodological quality was assessed using the original publication. Trial quality was evaluated using Cochrane review bias assessment risk criteria [11]. The GRADE approach was used to assess quality of evidence.

This included random sequence generation (selection bias), allocation concealment (selection bias), blinding of participants and personnel blinding (performance bias), outcome assessment blinding (detection bias), incomplete outcome data (attrition bias), selective reporting (reporting bias) and other biases. Possible ratings were ranked by risk: low (L); high (H); uncertain (U).

#### Statistical analyses

When appropriate, data extracted was combined for meta-analysis using Review Manager 5.3. Effect size was estimated and reported as the mean difference (MD) for continuous variables with a 95% confidence interval (CI). Weight was calculated in individual studies based on the inverse of variance. This study used a random-effects model for analyses due to expected heterogeneity of the studies selected. Study statistical homogeneity was assessed using a Cochran test and by examining the observed variances in effect sizes and residual variance. I^2^ was calculated to quantify heterogeneity. I^2^ > 50% was considered significant [11] . No statistical corrections were used to adjust for multiple analyses. We set the ultrasonic subgingival scaling as the experimental group and the manual subgingival scaling as the control group.

According to Cochrane reviews [11], in meta-analysis, studies with baseline changes as outcomes could be combined with those with final measurements as outcomes. In randomized trials, differences in mean values obtained from baseline changes were usually analyzed on the basis of final measurements and obtained the same effects. In this meta-analysis, baseline changes and final measurements from different studies were combined.

The reason of disagreement from previous studies about ultrasonic and manual subgingival scaling might be that they did not compare them in the same initial pocket depth. In other words, they did not control the variate of initial PPD. So, in this study, we established three subgroups based upon initial pocket depth: shallow (≤4 mm); medium (4-6 mm); and deep (≥6 mm), to eliminate the effect of the initial PPD to the result.

If a study had two initial PPD groups that could be included in one subgroup, data was combined to conform to the depth classification, using the formulas in Appendix 2 [11].

#### Sensitive analysis

Pre-planned sensitivity analysis had been done, analyzing the data in the same follow-up time and initial pocket depth: shallow (≤4 mm), medium (4-6 mm), or deep (≥6 mm). And we deleted a study each time and performed a new meta-analysis to see if the heterogeneity had changed significantly. If a study was deleted and the heterogeneity was significantly reduced, it was considered to be the main source of heterogeneity, needing further read and evaluation.

## Results

### Study selection

**Fig. 1:**
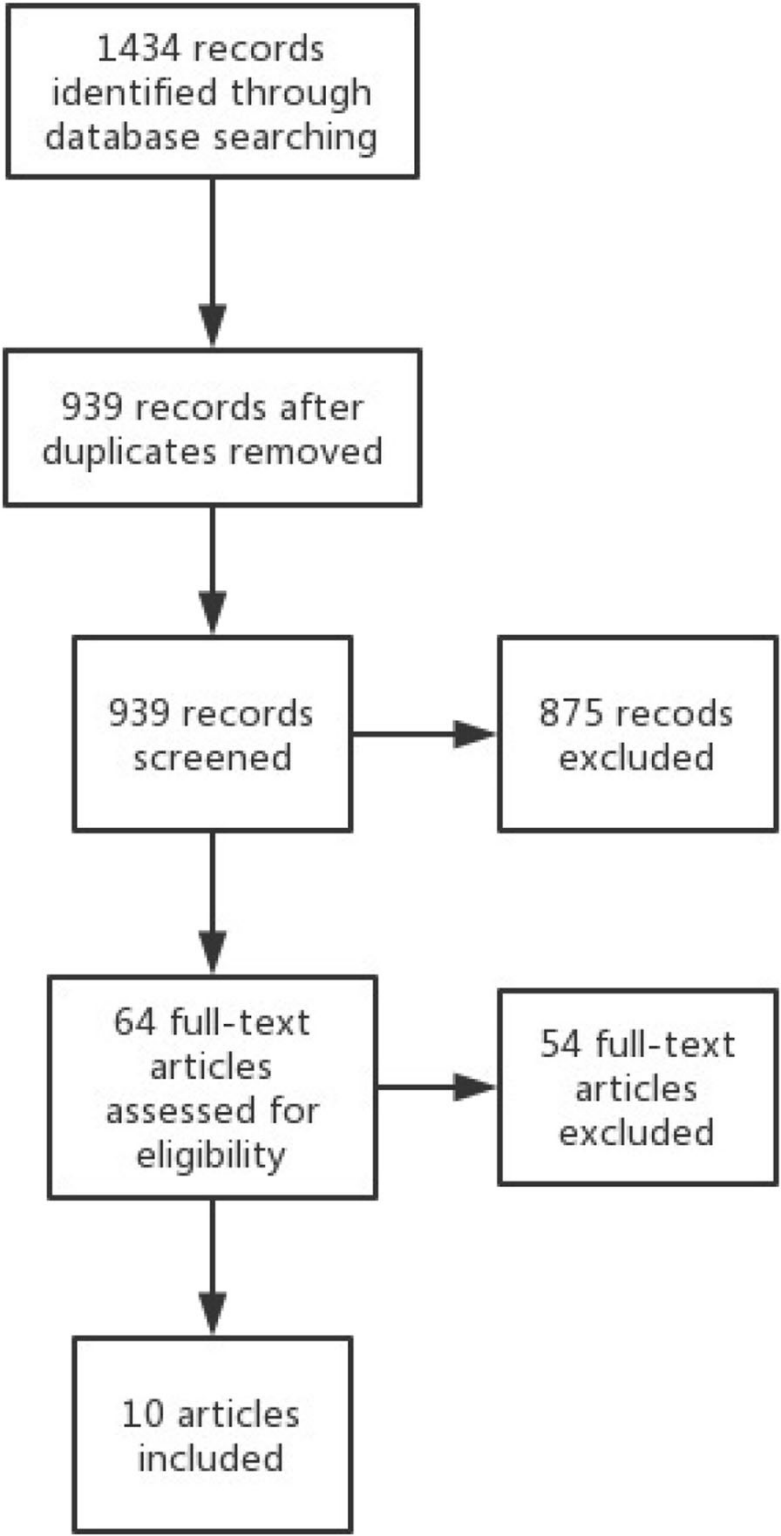
Selection process PRISMA flow chart

There were 1434 studies searched. References in selected papers were searched and no additional studies were located. After reading full texts, ten studies were finally selected. Process selection appears in Fig. 1.

There were 495 duplicates in the 1434 studies. 875 studies were ruled out after reading titles and abstracts. Another 43 studies were ruled out after reading the full text. There were 11 unavailable or awaiting classifications. Finally, 10 studies were included. Studies eventually included were from 2004 to 2015.

### Study characteristics

All research included were RCTs. Follow-up periods were 3 and 6 months. Characteristics of articles selected for primary outcomes appear in Table 1. Characteristics of articles selected for secondary outcomes appear in Table 2. Reasons for study exclusion appear in Table 3 [5, 6, 10, 24–63].

**Table 3:** Reasons for excluding studies

Study	Reasons
[24–32]	No UD-to-SRP comparisons.
[35–54]	Different PPD categories present in the same study or experimental group.
[56]	UD-SRP comparisons present and different PPD categories in the same study or experimental group.
[57, 58]	An *in vitro* study.
[10, 59]	Uncertain sample size.
[60, 61]	Study results were diagrams with unclear data.
[5]	There were treatments after the initial treatments during a follow-up period.
[62]	Follow-up was 24 months which is a long enough period for tissue changes affected factors other than the interventions. The injured tissue may have completely recovered spontaneously.
[63]	At each reassessment, study data was measured using different categories of PPD making it impossible to determine PPD change.

Limited information of 11 studies could be obtained from the publication. Inclusion, or exclusion could not be decided because of unobtainable full texts and unknown specific conditions. These appear in Appendix 3.

### Quality and risk of bias assessment

Bias analysis results for the studies appear in Appendix 4 and 5. Most studies did not have high bias risks. Funnel plots could not be done due to limited number of studies (< 10).

### Meta-analysis results of primary outcomes

The follow-up period lengths of the studies varied. Most were 3 and 6 months. This allowed for grouping into 3- and 6-months and reduced heterogeneity.

#### Outcome 1:PPD (Fig.2)

**Fig. 2:**
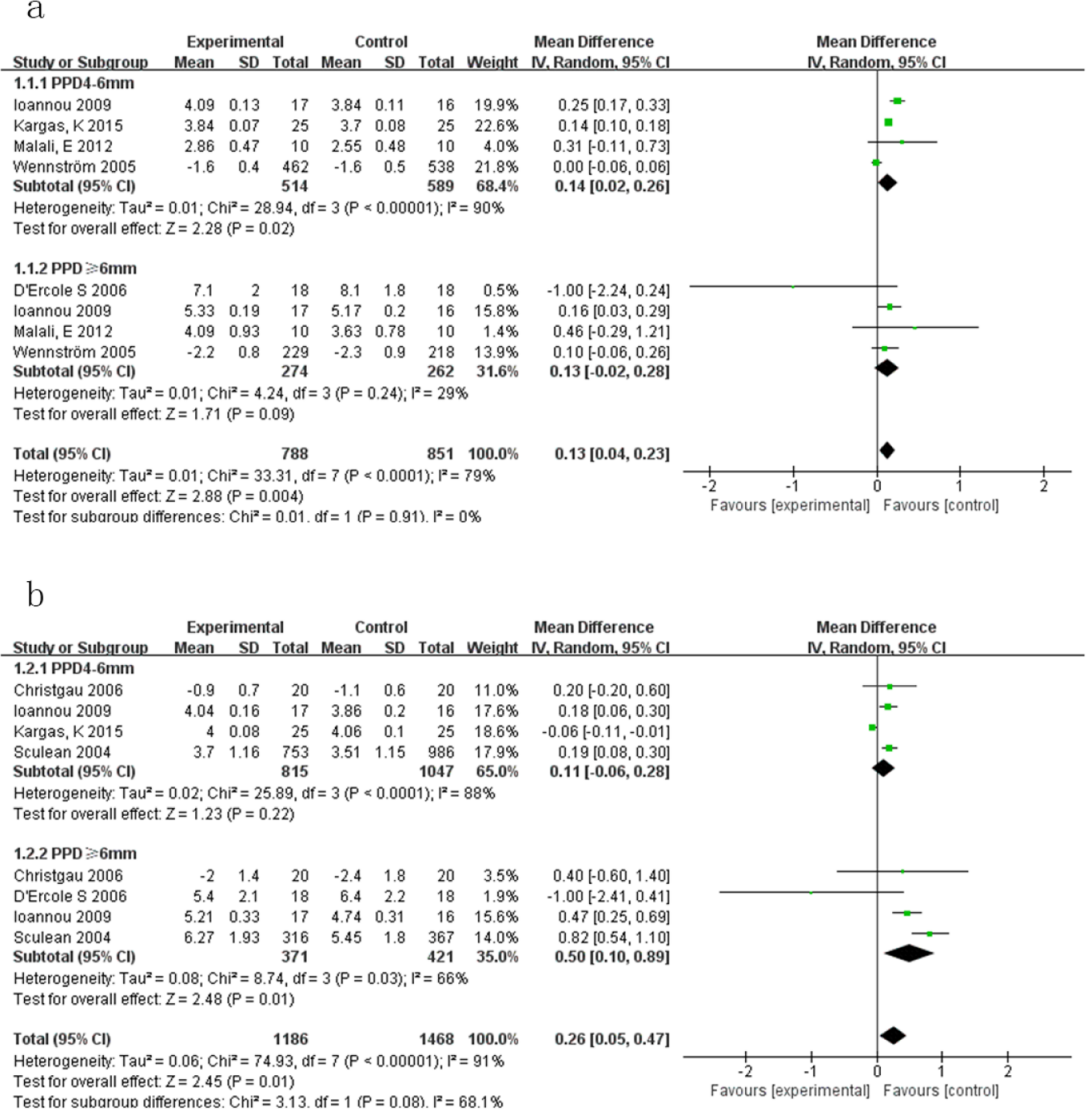
**a**. Forest plot comparing PPD at 3-months with manual subgingival scaling versus ultrasonic subgingival scaling of initial PPD > 4 mm in terms of the following: 1.1.1 initial PPD 4-6 mm; 1.1.2 initial PPD ≥ 6 mm. **b**. Forest plot comparing PPD at 6-months with manual subgingival scaling versus ultrasonic subgingival scaling of initial PPD > 4 mm in terms of the following: 1.2.1 initial PPD 4-6 mm; 1.2.2 initial PPD ≥ 6 mm

1. 3 months: (Fig. 2a)

- Initial PPD ≤4 mm
  Only one met the criteria, which reported no statistical differences between ultrasonic and manual subgingival scaling [12].
- Initial PPD > 4 mm
  When initial PPD was medium, differences between ultrasonic and manual subgingival scaling were statistically significant. PPD reduction after manual subgingival scaling was greater than ultrasonic instruments (MD 0.14, 95% CI [0.02, 0.26], *P* = 0.02). Heterogeneity was great (Tau^2^ = 0.01; Chi^2^ = 28.94, df = 3 (*P* < 0.00001); I^2^ = 90%) [12–15].
  When initial PPD was deep, heterogeneity was acceptable (Tau^2^ = 0.01; Chi^2^ = 4.24, df = 3 (*P* = 0.24); I^2^ = 29%). PPD reduction after the two treatments were not statistically significant (MD 0.13, 95%CI [− 0.02, 0.28], *P* = 0.09) [12–14, 16].

(2) 6 months: (Fig. 2b)

- Initial PPD ≤4 mm

Two studies [12, 17] met the criteria as they used baseline changes as outcomes and one baseline changes value of 0. A meta-analysis could not be done using the two studies. They both reported no statistical differences between ultrasonic and manual subgingival scaling.

b) Initial PPD > 4 mm

When initial PPD was medium, differences between ultrasonic and manual subgingival scaling were not statistically significant (MD 0.19, 95%CI [0.11, 0.27], *P* = 0.22). Heterogeneity was large (Tau^2^ = 0.02; Chi^2^ = 25.89, df = 3 (*P* < 0.0001); I^2^ = 88%) [12, 15, 17, 18].

When initial PPD was deep, heterogeneity was also great (Tau^2^ = 0.08; Chi^2^ = 8.74, df = 3 (*P* = 0.03); I^2^ = 66%). PPD reduction after manual subgingival scaling was greater than ultrasonic instruments with a statistically significant difference (MD 0.50, 95%CI [0.10, 0.89], *P* = 0.01) [12, 16–18].

#### Outcome 2:CAL (Fig. 3)

**Fig. 3:**
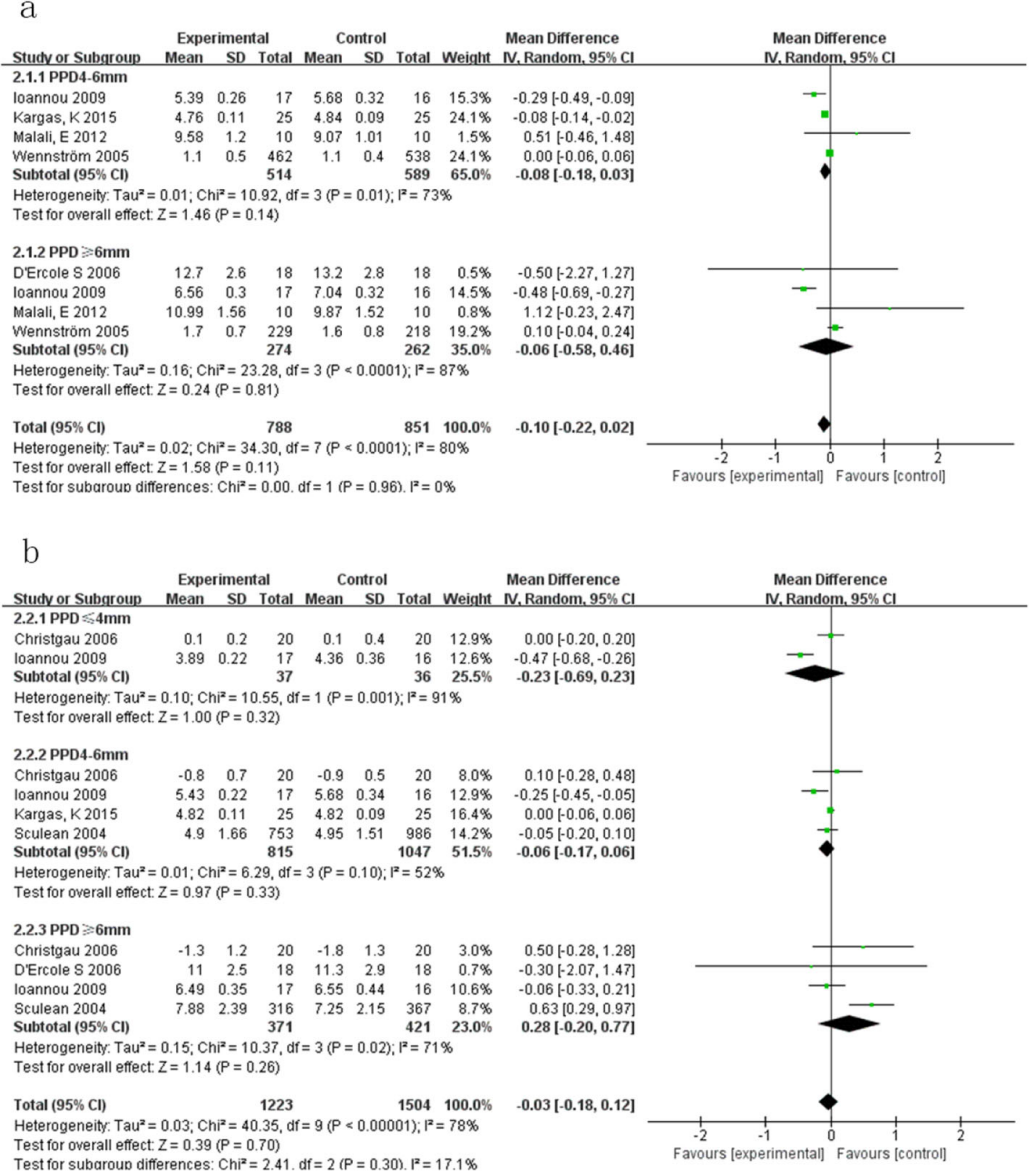
**a**. Forest plot comparing CAL at 3-months with manual subgingival scaling versus ultrasonic subgingival scaling of initial PPD > 4 mm in terms of the following: 2.1.1 initial PPD4-6 mm; 2.1.2 initial PPD ≥ 6 mm. **b**. Forest plot comparing CAL at 6-months with manual subgingival scaling l versus ultrasonic subgingival scaling of initial PPD were shallow in terms of the following: 2.2.1 initial PPD ≤ 4 mm;2.2.2 initial PPD4-6 mm; 2.2.3 initial PPD ≥ 6 mm

1. 3 months: (Fig. 3a)

- Initial PPD ≤4 mm

Only one met the criteria, reporting no statistically significant difference between the two methods [12].

b) Initial PPD > 4 mm

When initial PPD was medium, differences between ultrasonic and manual subgingival scaling were not statistically significant (MD -0.08, 95%CI [− 0.18, 0.03], *P* = 0.14). Heterogeneity was great (Tau^2^ = 0.01; Chi^2^ = 10.92, df = 3 (P = 0.01); I^2^ = 73%) [12–15].

When initial PPD was deep, difference between ultrasonic and manual subgingival scaling was not statistically significant (MD -0.06, 95%CI [− 0.58, 0.46], *P* = 0.81). Heterogeneity was also high (Tau^2^ = 0.16; Chi^2^ = 23.28, df = 3 (*P* < 0.0001); I^2^ = 87%) [12–14, 16].

(2) 6 months: (Fig. 3b)

- Initial PPD ≤4 mm

The heterogeneity of the two studies was too large (Tau^2^ = 0.10; Chi^2^ = 10.55, df = 1 (*P* = 0.001); I^2^ = 91%) for a meta-analysis to be performed. They both indicated no statistically significant differences between ultrasonic and manual instruments [12, 17].

b) Initial PPD > 4 mm

No statistically significant differences were found between the ultrasonic and manual subgingival scaling when initial PPD was medium (MD -0.06, 95%CI [− 0.17, 0.06], *P* = 0.33). Heterogeneity was slightly great (Tau^2^ = 0.01; Chi^2^ = 6.29, df = 3 (*P* = 0.10); I^2^ = 52%) [12, 15, 17, 18].

At deep pocket depth, differences between the two were not statistically significant (MD 0.28, 95%CI [− 0.20, 0.77], *P* = 0.26). Heterogeneity was large (Tau^2^ = 0.15; Chi^2^ = 10.37, df = 3 (*P* = 0.02); I^2^ = 71%) [12, 16–18].

### Secondary outcome measures

1. GR: Sculean et al. [18] indicated no statistical differences studying single or multiply-root teeth between ultrasonic and manual subgingival scaling at 6-months when initial PPD was deep. Kargas et al. [15] noted that, at medium depths, there were no statistical differences between ultrasonic and manual subgingival scaling at either 3 or 6-months.
2. BOP**:** Christgau et al. [17] found that at 6-months, manual subgingival scaling showed greater BOP reduction at initial deep pocket depth compared to ultrasound.
3. Residual dental calculus: Schwarz et al. [19] indicated that for single-root teeth at deep initial depth, ultrasonic subgingival device was superior to manual instruments in removing subgingival dental calculus. Yukna et al. [20] found no statistical differences in residual dental calculus rates between ultrasonic and manual subgingival scaling with initial PPD at 5-6 mm, 7-8 mm or > 9 mm. Gellin et al. [21] found no statistical differences in dental calculus clearance rates between the two methods when initial PPD was 0-3 mm, 4-5 mm, or, 6-12 mm. When ultrasonic subgingival scaling was combined with manual instruments, the effectiveness was superior to either ultrasonic or manual instruments individually [21].

## Discussion

### Sensitivity analysis

#### Outcome: PPD

At 3-months, at medium depth, heterogeneity was great (I^2^ = 90%, Fig. 2a). After sensitivity analysis, four studies were found highly heterogeneous to each other and were unsuitable for meta-analysis. After a bias analysis, the heterogeneity source was thought to be: (1) small number of studies; (2) the fact that tissue healing took time and early probing disrupted attachment gains. At 3-months, PPD and CAL reductions were unstable, causing large heterogeneity.

**Fig. 4:**
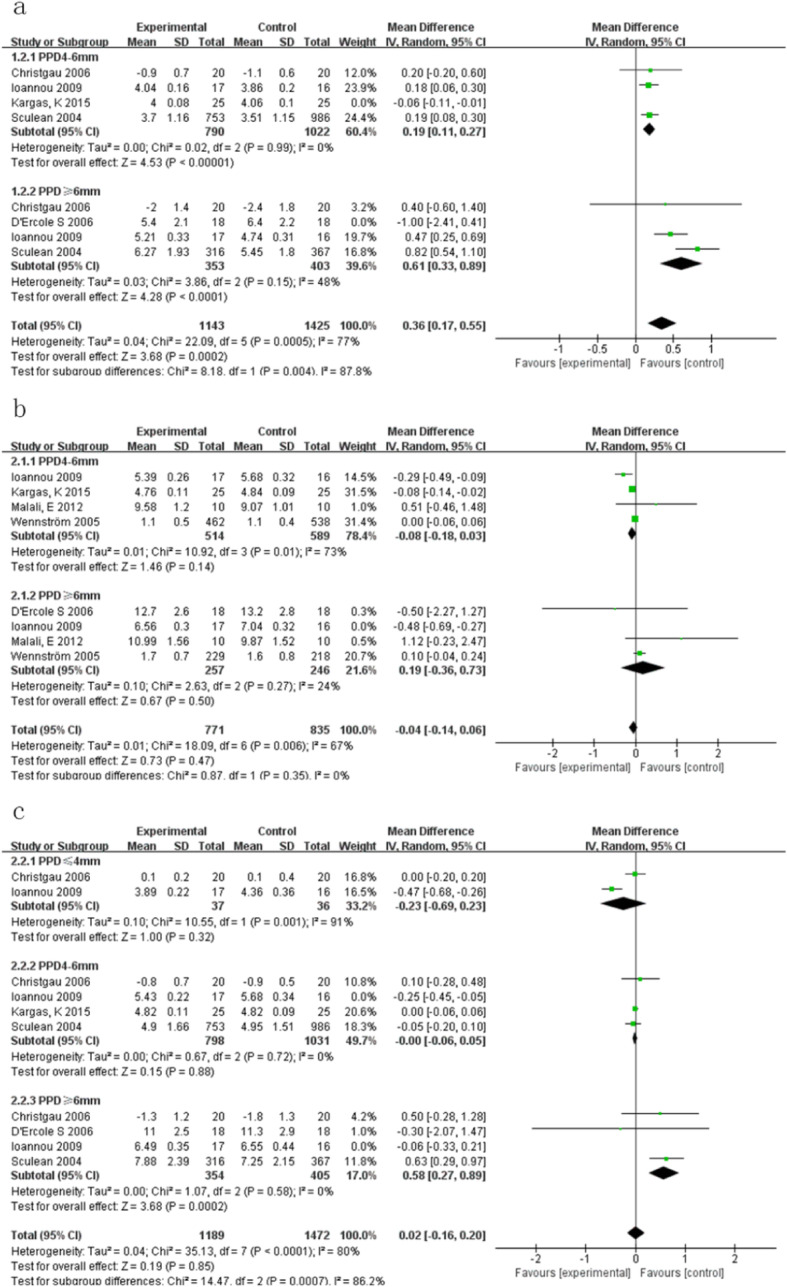
**a**. Sensitivity analysis of PPD at 6-months in terms of the following: 1.2.1 initial PPD 4-6 mm; 1.2.2 initial PPD ≥6 mm. **b**. Sensitivity analysis of CAL at 3-months in terms of the following: 2.1.1 initial PPD 4-6 mm; 2.1.2 initial PPD ≥6 mm. **c**. Sensitivity analysis of CAL at 6-months in terms of the following: 2.2.1 initial PPD ≤ 4 mm; 2.2.2 initial PPD 4-6 mm; 2.2.3 initial PPD ≥6 mm

At medium depth, 6-months, Kargas et al. [15] was found to have significant heterogeneity. After excluded, heterogeneity decreased to 0% (Tau^2^ = 0.00; Chi^2^ = 0.02, df = 2 (*P* = 0.99); I^2^ = 0%). The results showed statistically significant differences between manual and ultrasound groups. PPD reduction after manual subgingival scaling was greater than ultrasonic subgingival scaling (MD 0.19,95%CI [0.11, 0.27], *P* < 0.00001). Compared with the other three studies, only non-smokers were included in this study, which might be the reason for heterogeneity (Fig. 4a).

When initial PPD was deep at 6 months, D’Ercole [16] was a major origin of heterogeneity. After exclusion, heterogeneity decreased to (Tau^2^ = 0.03; Chi^2^ = 3.86, df = 2 (*P* = 0.15); I^2^ = 48%) (Fig. 4a).

#### Outcome:CAL

When initial PPD was medium, at 3-months follow-up, the heterogeneity of CAL was high (Tau^2^ = 0.01; Chi^2^ = 10.92, df = 3 (*P* = 0.01); I^2^ = 73%). According to a sensitivity analysis, four studies were highly heterogeneous with each other, making them unsuitable for meta-analysis (Fig. 4b). The heterogeneity source was also thought to be: (1) too few studies; (2) tissue healing took time and early intervening probing may damage attachment gain. When the follow-up period was only 3 months, CAL were unstable which caused great heterogeneity.

Heterogeneity was also large in the following three groups: 1) deep pocket, at 3-months follow-up; 2) medium pocket, at 6-months follow-up; 3) deep pocket, at 6-months follow-up. After excluding Ioannou 2009 [12], heterogeneity decreased from: 87, 52, 71 to 24%, 0, 0%. This study was the only one in which 50% of the patients were smokers, while other papers were unclear about the ratio of smokers or had a small number of smokers, which might be the reason for heterogeneity. After exclusion, at deep pocket depth of a 6-months follow-up, after manual subgingival scaling, CAL reduction was more than ultrasonic subgingival scaling and were statistically different. (MD 0.58, 95%CI [0.27, 0.89], *P* = 0.0002) (Fig. 4b, Fig. 4c).

In the same study, heterogeneity of PPD and CAL was much greater at 3-months than at 6-months follow-up. The influence was quite apparent at medium PPD, 3-months. At deep initial depths, either 3-months or 6-months, heterogeneity was acceptable. The reason could be that the tissue took time to heal. In deep pocket, tissue contacted and attached to the bone better, resulting in shorter healing time and more stable condition within 3 months. At medium pocket depths, tissue did not contact bone as readily as at deeper pockets, so healing time was longer. Probing too early in healing process may damage tissue and influence attachment gain, and led to unstable results.

### Quality of evidence

After evaluating the quality of data with the GRADE system, the following results were obtained: the data of PPD at 3-months and 6-months follow-up of shallow pocket, and CAL at 3-months follow-up of shallow pocket was of very low quality; the data of PPD at 3-months follow-up of medium pocket, CAL at 3-months follow-up of medium and deep pocket and at 6-months follow-up of shallow pocket was of low quality; the data of PPD at 3-months follow-up of medium pocket, at 6-months follow-up of medium and deep pocket and CAL at 6-months follow-up of medium and deep pocket was of moderate quality. Details were given in the Appendix 6. The data of shallow pocket mostly was of very low quality because of too small sample size and publication bias, so that we could not draw a reliable conclusion and more studies are required. The data of 3-months follow-up was of low or very low quality, which might due to the fact that tissue healing took time and early probing disrupted attachment gains. We thought 3-months follow-up was too short and we cannot draw reliable conclusions according to the data of it. The data of PPD and CAL at 6-months follow-up for the medium and deep pocket groups was of moderate quality. Based on the above, we thought the conclusion should be drawn according to the data of 6-months follow-up of medium and deep pocket groups.

According to the above analysis, different indicators showed statistical significance between ultrasonic and manual subgingival scaling, which indicated the different effectiveness in clinic after the treatment of ultrasonic and manual instruments.

In addition, in shallow pocket, CAL increased after both ultrasonic and manual subgingival scaling, which might be resulted from junctional epithelium attachment damage [17]. We have also found it clinically. Therefore, manual subgingival scaling is not recommended when PPD is less than 4 mm. In clinical practice, when PPD is less than 4 mm and there is symptom such as bleeding on probing or subgingival dental calculus, ultrasonic subgingival working tip can be used for deep cleaning.

In terms of GR, at medium or deep PPD, there was no differences between manual and ultrasonic subgingival scaling, whatever the roots were single or multiple [15, 18].

BOP results of one study [17] showed, at 6-months follow-up, more BOP reduction at deep depths after manual scaling than ultrasound.

Residual calculus provided different results. Two studies [20, 21] indicated that, regardless of depth, there were no statistical differences in calculus clearance rates between ultrasound and manual treatment. Schwarz [19] indicated when PPD was deep, for a single-root tooth, ultrasonic dental calculus removal was more effective than manual subgingival scaling.

Above all, ultrasonic subgingival scaling is an efficient non-surgical treatment [7], yet manual subgingival scaling is also essential and cannot be replaced by ultrasonic method.

According to the newly-released 2018 periodontitis classifications, there are something about it:

1. Different economic and health care developments between developed and developing countries make different influences on periodontitis [22, 23]. Primary CAL in developing countries was three times of developed countries [23]. Only one study [13] involved a developing country (Turkey). Whether the conclusions reached in this paper apply to developing countries is unknown.
2. Smoking is confirmed as affecting progress of periodontitis and was considered in the new classification [22]. Most of the included studies chose patients according to the 1999 classification and did not consider the impact of smoking, which may lead to heterogeneity.

### Summary

#### Significance to clinical practice

1. Combining our above analysis and quality of evidence, we believed that only 6-months follow-up results could be used to reach following conclusions:
  - When initial PPD was shallow, no conclusions were drawn due to the limited number of studies.
  - When initial PPD was medium, PPD reductions proved that manual subgingival scaling was superior. CAL and GR results showed no statistical differences. More studies are needed before any conclusion can be drawn.
  - When initial PPD was deep, manual subgingival scaling was superior in terms of PPD, CAL and BOP results, while GR results showed no statistical differences. This conclusion also needs more study because of the limited number of studies.
2. In terms of residual dental calculus, there was no conclusion could be drawn.

#### Significance to research

1. Inclusion and exclusion criteria could refer to the new classification to reduce bias;
2. Studies should consider other indicators such as BOP, PI, and GI, bacterial changes, when comparing in different PPDs;
3. More studies are needed in developing countries;
4. Single and multiple root teeth should be measured separately;
5. Further studies should enlarge sample sizes to improve credibility;
6. Inclusion or exclusion criteria for smokers should be standardized.
7. Studies with a follow-up period of 6 months or longer are suggested to determine reliable results.

## Conclusions

Ultrasonic subgingival scaling is an efficient non-surgical treatment of periodontitis. However, when initial PPD was 4-6 mm, PPD reduction proved manual subgingival scaling was superior, but CAL results showed no statistical differences between the two means. When initial PPD was ≥6 mm, PPD and CAL reductions suggested that manual subgingival scaling was superior. Manual subgingival scaling is significant and cannot be completely replaced by ultrasonic subgingival scaling. We suggest, when initial PPD is medium or deep, using ultrasonic and manual subgingival instruments together.

## Supplementary information

**Additional file 1 Appendix 1**. Search strategy used in PubMed/MEDLINE. **Appendix 2**. Data merger formulas. **Appendix 3**. Characteristics of studies awaiting classification. **Appendix 4**. Risk of bias graph. **Appendix 5**. Risk of bias summary. **Appendix 6**. GRADE quality of evidence.

## Data Availability

We declared that materials described in the manuscript, including all relevant raw data, will be freely available to any scientist wishing to use them for non-commercial purposes, without breaching participant confidentiality. All data generated or analyzed during this study are included in this published article (and its supplementary information files).

## Abbreviations

AL: Attachment level
BOP: Bleeding on probing
CAL: Clinical attachment loss
GR: Gingival recession
MD: Mean depth
PPD: Probing pocket depth
