# Supplementary material for: Treating periodontitis-a systematic review and meta-analysis comparing ultrasonic and manual subgingival scaling at different probing pocket depths
Source: BMC Oral Health. 2020 Jun 25;20:176. doi: 10.1186/s12903-020-01117-3 (PMC7318456; doi:10.1186/s12903-020-01117-3)
Supplement: Supplementary file 1 — Additional file 1 Appendix 1. Search strategy used in PubMed/MEDLINE. Appendix 2. Data merger formulas. Appendix 3. Characteristics of studies awaiting classification. Appendix 4. Risk of bias graph. Appendix 5. Risk of bias summary. Appendix 6. GRADE quality of evidence. [file 12903_2020_1117_MOESM1_ESM.docx]

**Appendices**

Appendix 1. Search strategy used in PubMed/MEDLINE

|  | Search terms |
| --- | --- |
| No. 4 | No 1 and No 2 and No 3 |
| No. 3 | (((hand*) OR hand) OR manual*) |
| No. 2 | (((((((((((((sonic*) OR ultrasonic*) OR oscillat*) OR reciprocat*) OR rotat*) OR diamond*) OR air*AND abras*) OR power-driven*) OR machine-driven*)) OR (("Ultrasonic Therapy"[Mesh]) OR (((Ultrasonic Therapy) OR Therapies, Ultrasonic) OR Ultrasonic Therapies))) OR (("Lithotripsy"[Mesh]) OR (((((((((((((((((((((((((((((((Lithotripsies) OR Litholapaxy) OR Litholapaxies) OR Percutaneous Ultrasonic Lithotripsy) OR Lithotripsies, Percutaneous Ultrasonic) OR Lithotripsy, Percutaneous Ultrasonic) OR Percutaneous Ultrasonic Lithotripsies) OR Ultrasonic Lithotripsies, Percutaneous) OR Ultrasonic Lithotripsy, Percutaneous) OR Ultrasonic Lithotripsy) OR Lithotripsies, Ultrasonic) OR Lithotripsy, Ultrasonic) OR Ultrasonic Lithotripsies) OR Extracorporeal Shockwave Lithotripsy) OR Extracorporeal Shockwave Lithotripsies) OR Lithotripsies, Extracorporeal Shockwave) OR Lithotripsy, Extracorporeal Shockwave) OR Shockwave Lithotripsies, Extracorporeal) OR Shockwave Lithotripsy, Extracorporeal) OR Electrohydraulic Shockwave Lithotripsy) OR Electrohydraulic Shockwave Lithotripsies) OR Lithotripsies, Electrohydraulic Shockwave) OR Lithotripsy, Electrohydraulic Shockwave) OR Shockwave Lithotripsies, Electrohydraulic) OR Shockwave Lithotripsy, Electrohydraulic) OR ESWL (Extracorporeal Shockwave Lithotripsy)) OR ESWLs (Extracorporeal Shockwave Lithotripsy)) OR Noninvasive Litholapaxy) OR Litholapaxies, Noninvasive) OR Litholapaxy, Noninvasive) OR Noninvasive Litholapaxies))) OR (("Ultrasonic Surgical Procedures"[Mesh]) OR (((((((((Procedure, Ultrasonic Surgical) OR Procedures, Ultrasonic Surgical) OR Surgical Procedure, Ultrasonic) OR Surgical Procedures, Ultrasonic) OR Ultrasonic Surgical Procedure) OR Ultrasonic Surgery) OR Surgeries, Ultrasonic) OR Surgery, Ultrasonic) OR Ultrasonic Surgeries)))) |
| No. 1 | (((((((((((((("Chronic Periodontitis"[Mesh]) OR ((((((((((Chronic Periodontitides) OR Periodontitides, Chronic) OR Periodontitis, Chronic) OR Adult Periodontitis) OR Adult Periodontitides) OR Periodontitides, Adult) OR Periodontitis, Adult) OR Periodontitides) OR Pericementitis) OR Pericementitides))) OR (("Periodontal Pocket"[Mesh]) OR (((Pocket, Periodontal) OR Periodontal Pockets) OR Pockets, Periodontal))) OR (("Dental Plaque"[Mesh]) OR Plaque, Dental)) OR (("Periodontal Debridement"[Mesh]) OR (((((((((((((Debridement, Periodontal) OR Debridements, Periodontal) OR Periodontal Debridements) OR Nonsurgical Periodontal Debridement) OR Debridement, Nonsurgical Periodontal) OR Debridements, Nonsurgical Periodontal) OR Nonsurgical Periodontal Debridements) OR Periodontal Debridement, Nonsurgical) OR Periodontal Debridements, Nonsurgical) OR Periodontal Pocket Debridement) OR Debridement, Periodontal Pocket) OR Debridements, Periodontal Pocket) OR Periodontal Pocket Debridements))) OR (("Dental Calculus"[Mesh]) OR ((Tartar) OR Calculus, Dental))) OR (("Dental Scaling"[Mesh]) OR (((((((((Subgingival Scaling) OR Scaling, Subgingival) OR Scalings, Root) OR Root Scalings) OR Root Scaling) OR Scaling, Root) OR Supragingival Scaling) OR Scaling, Supragingival) OR Scaling, Dental))) OR (("Root Planing"[Mesh]) OR ((Planing, Root) OR Planings, Root))) OR (("Subgingival Curettage"[Mesh]) OR (((((((((((((Curettage, Subgingival) OR Curettages, Subgingival) OR Subgingival Curettages) OR Gingival Curettage) OR Curettage, Gingival) OR Curettages, Gingival) OR Gingival Curettages) OR Periodontal Epithelial Debridement) OR Debridement, Periodontal Epithelial) OR Debridements, Periodontal Epithelial) OR Epithelial Debridement, Periodontal) OR Epithelial Debridements, Periodontal) OR Periodontal Epithelial Debridements))) OR (("Dental Prophylaxis"[Mesh]) OR Prophylaxis, Dental)) OR (("Dental Deposits"[Mesh]) AND ((((Materia Alba) OR Deposits, Dental) OR Dental Deposit) OR Deposit, Dental)))) |

Appendix 2. Data merger formulas

|  | Group 1 | Group 2 | Combined groups |
| --- | --- | --- | --- |
| Sample size | 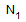 | 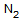 | 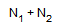 |
| Mean | 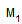 | 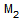 | 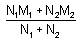 |
| SD | 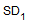 | 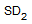 | 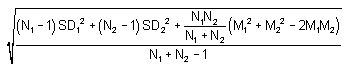 |

Appendix 3. Characteristics of studies awaiting classification

| title | first author | year |
| --- | --- | --- |
| Debris forced through apical foramina by ultrasonic and hand instrumentation | Wright, G. L. | 1993 |
| A clinical and SEM evaluation of the efficiency of sofscale gel and hand scaling and hand scaling alone | Thomas, K. | 2002 |
| Effects of curet and ultrasonics on root surfaces | Cross-Poline, G. N. | 1995 |
| A clinical evaluation of hand and ultrasonic instruments on subgingival debridement. 1. With unmodified and modified ultrasonic inserts | Dragoo, M. R. | 1992 |
| Periodontal healing after treatment with either the Titan-S sonic scaler or hand instruments | Laurell, L. | 1988 |
| Calculus removal and loss of tooth substance in response to different periodontal instruments. A scanning electron microscope study | Lie, T. | 1977 |
| A comparative study between different techniques in non-surgical periodontal treatment | Forabosco, A. | 2006 |
| A quadrant-design trial of four therapeutic modalities in chronic moderate periodontitis | Nonhoff, J. | 2006 |
| Study of the influence of different instruments for removing dental deposits on tooth surface | Kuchumova, E. D. | 2006 |
| Scanning eletronmicroscopical studies of the root surface following the use of various hand and ultrasonic instruments | Diedrich, P. | 1975 |
| Effect of manual and ultrasonic instrumentation on the radicular surface | Caffesse, R. G. | 1973 |

Appendix 4. Risk of bias graph


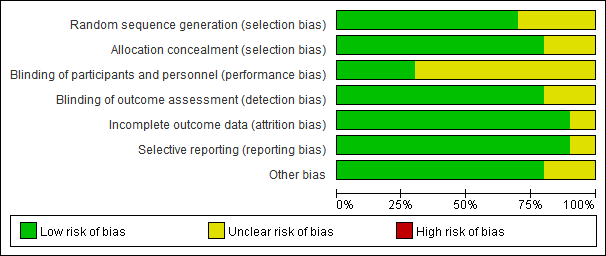


Appendix 5. Risk of bias summary


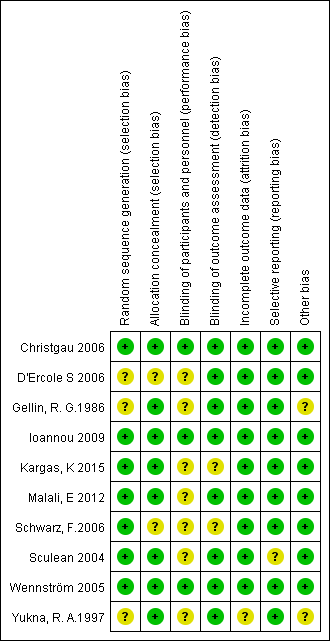


Appendix 6. GRADE quality of evidence

**Primary outcome: PPD**

| **Quality assessment** | | | | | | | **No of patients** | | **Effect** | **Quality** | **Importance** |
| --- | --- | --- | --- | --- | --- | --- | --- | --- | --- | --- | --- |
| **No of studies** | **Design** | **Risk of bias** | **Inconsistency** | **Indirectness** | **Imprecision** | **Other considerations** | **Experiment** | **Control** | **Mean Difference (95% CI)** |  |  |
| **3month - PPD≤4mm** | | | | | | | | | | | |
| 1 | randomised trials | serious^1^ | no serious inconsistency | no serious indirectness | serious^2^ | reporting bias^3^ | 17 | 16 | MD 0.01 lower (0.07 lower to 0.05 higher) | ⊕OOO VERY LOW | CRITICAL |
| **3month - PPD 4-6mm** | | | | | | | | | | | |
| 4 | randomised trials | serious^1,4^ | serious^5^ | no serious indirectness | no serious imprecision | none | 514 | 589 | MD 0.14 higher (0.02 to 0.26 higher) | ⊕⊕OO LOW | CRITICAL |
| **3month - PPD≥6mm** | | | | | | | | | | | |
| 4 | randomised trials | serious^1^ | no serious inconsistency | no serious indirectness | no serious imprecision | none | 274 | 262 | MD 0.13 higher (0.02 lower to 0.28 higher) | ⊕⊕⊕O MODERATE | CRITICAL |
| **6month - PPD≤4mm** | | | | | | | | | | | |
| 2 | randomised trials | no serious risk of bias | serious^6^ | no serious indirectness | serious^2^ | reporting bias^3^ | 37 | 36 | MD 0.06 higher (0.03 lower to 0.15 higher) | ⊕OOO VERY LOW | CRITICAL |
| **6month - PPD4-6mm** | | | | | | | | | | | |
| 3 | randomised trials | serious^4^ | no serious inconsistency | no serious indirectness | no serious imprecision | none | 790 | 1022 | MD 0.19 higher (0.11 to 0.27 higher) | ⊕⊕⊕O MODERATE | CRITICAL |
| **6month - PPD≥6mm** | | | | | | | | | | | |
| 3 | randomised trials | serious^4^ | no serious inconsistency | no serious indirectness | no serious imprecision | none | 353 | 403 | MD 0.61 higher (0.33 to 0.89 higher) | ⊕⊕⊕O MODERATE | CRITICAL |

^1^ Too short follow-up period
^2^ Sample size is small
^3^ No large sample size data was found
^4^ Blinding is not clear in one study that weighs a lot
^5^ Minimal or no overlap of confidence intervals (CI)
^6^ Heterogeneity cannot be assessed

**Primary outcome: CAL**

| **Quality assessment** | | | | | | | **No of patients** | | **Effect** | **Quality** | **Importance** |
| --- | --- | --- | --- | --- | --- | --- | --- | --- | --- | --- | --- |
| **No of studies** | **Design** | **Risk of bias** | **Inconsistency** | **Indirectness** | **Imprecision** | **Other considerations** | **Experiment** | **Control** | **Mean Difference (95% CI)** |  |  |
| **3month - PPD≤4mm** | | | | | | | | | | | |
| 1 | randomised trials | serious^1^ | no serious inconsistency | no serious indirectness | serious^2^ | reporting bias^3^ | 17 | 16 | MD 0.65 lower (0.86 to 0.44 lower) | ⊕OOO VERY LOW | CRITICAL |
| **3month - PPD4-6mm** | | | | | | | | | | | |
| 4 | randomised trials | serious^1,4^ | serious^5^ | no serious indirectness | no serious imprecision | none | 514 | 589 | MD 0.08 lower (0.18 lower to 0.03 higher) | ⊕⊕OO LOW | CRITICAL |
| **3month - PPD≥6mm** | | | | | | | | | | | |
| 3 | randomised trials | serious^1^ | no serious inconsistency | no serious indirectness | serious^6^ | none | 257 | 246 | MD 0.19 higher (0.36 lower to 0.73 higher) | ⊕⊕OO LOW | CRITICAL |
| **6month - PPD ≤4mm** | | | | | | | | | | | |
| 2 | randomised trials | no serious risk of bias | serious^2,7^ | no serious indirectness | serious^2,6^ | none | 37 | 36 | MD 0.23 lower (0.69 lower to 0.23 higher) | ⊕⊕OO LOW | CRITICAL |
| **6month - PPD4-6mm** | | | | | | | | | | | |
| 3 | randomised trials | serious^4^ | no serious inconsistency | no serious indirectness | no serious imprecision | none | 798 | 1031 | MD 0 higher (0.06 lower to 0.05 higher) | ⊕⊕⊕O MODERATE | CRITICAL |
| **6month - PPD≥6mm** | | | | | | | | | | | |
| 3 | randomised trials | serious^4^ | no serious inconsistency | no serious indirectness | no serious imprecision | none | 354 | 405 | MD 0.58 higher (0.27 to 0.89 higher) | ⊕⊕⊕O MODERATE | CRITICAL |

^1^ Too short follow-up period
^2^ Sample size is small
^3^ No large sample size data was found
^4^ Blinding is not clear in one study that weighs a lot
^5^ Minimal or no overlap of confidence intervals (CI)
^6^ Large span of confidence intervals (CI)
^7^ Unexplainable heterogeneity
